# Association between abortion restrictiveness and suicidality among birthing people in the United States 2010 to 2020

**DOI:** 10.3389/frph.2025.1553493

**Published:** 2025-08-29

**Authors:** I. S. Platt, K. Zivin, Z. Xiaosong, A. Tilea, E. Miller, A. Widner, A. Courant, S. V. Hall, A. Schroeder, V. K. Dalton

**Affiliations:** ^1^ Department of Health Policy and Management, The George Washington University Milken Institute School of Public Health, Washington, DC, United States; ^2^ Department of Psychiatry, University of Michigan Medical School, Ann Arbor, MI, United States; ^3^ Program on Women’s Healthcare Effectiveness Research, Department of Obstetrics and Gynecology, University of Michigan Medical School, Ann Arbor, MI, United States; ^4^ Department of Obstetrics and Gynecology, University of Michigan Medical School, Ann Arbor, MI, United States; ^5^ Department of Health Policy and Management, University of Michigan School of Public Health, Ann Arbor, MI, United States; ^6^ VA Ann Arbor Healthcare System, Ann Arbor, MI, United States; ^7^ Institute for Healthcare Policy and Innovation, University of Michigan, Ann Arbor, MI, United States

**Keywords:** suicidality, abortion policy, perinatal mental health, United States, mental health

## Abstract

**Introduction:**

Prior research found that suicidality increased among commercially insured birthing people between 2006 and 2017. The 2022 *Dobbs v. Jackson Women's Health Organization* decision overturned *Roe v. Wade* and made obtaining an abortion more difficult across the United States, which may have negative mental health effects among commercially insured birthing people.

**Methods:**

We conducted a cross-sectional analysis using mixed-effects logistic regression models to quantify the relationship between state-level abortion restrictions and a diagnosis of suicidality or self-harm in the 12 months before or after delivery among individuals with employer-sponsored health insurance in the United States who delivered between 2010 and 2020.

**Results:**

Of the 610,177 deliveries in our final analytic cohort, birthing people residing in states with high abortion restrictiveness were younger (12.8% of birthing people were ages 15–24 compared to 7.8% in low restriction states) and more likely to be Black (10.4% compared to 6.1%). Birthing people living in states with high abortion restrictiveness were more likely to experience suicidality than birthing people living in states with low abortion restrictiveness [odds ratio (OR): 1.5; 95% CI: 1.2, 1.8; *p* = 0.0012]. When controlling for age, state abortion restrictiveness was not significantly associated with suicidality [adjusted odds ratio (adjOR): 1.2; 95% CI: 1.0, 1.4; *p* = 0.0603], and birthing people ages 15–24 were substantially more likely than birthing people ages 35–44 to experience suicidality (adjOR: 7.3; 95% CI: 6.5, 8.2; *p* < 0.001).

**Conclusion:**

In the years prior to the *Dobbs* decision, commercially insured birthing people in states with high abortion restrictiveness experienced a growing mental health crisis, when compared to those in low restriction states. These differences are associated with differences in demographic characteristics, such as age and race. As researchers continue to monitor health outcomes related to the recent enactment of the most severe category of restriction (e.g., bans), these findings remain crucial to recognize and account for in further studies.

## Introduction

The United States (U.S.) has the highest maternal mortality rate of any high-income nation, with severe racial and ethnic inequities among those experiencing maternal mortality (1, 2). In 2022, 22.3 maternal deaths per 100,000 live births occurred in the U.S., with Black women experiencing a substantially higher rate of 49.5 deaths per 100,000 live births (2). While maternal deaths due to direct obstetrical causes have decreased (3), deaths by suicide remain high (4).

Behavioral health conditions, including depression, anxiety, and substance use disorders, now represent a leading cause of preventable maternal deaths in the U. S (5). Between 2007 and 2018, almost two-thirds of preventable maternal mental health deaths occurred by suicide (5). Suicidality, a precursor to completed suicide, increased three-fold among commercially insured birthing people during the 12 months before or after childbirth between 2006 and 2017. (6) Researchers found that suicidality increased most sharply among individuals ages 15–18 and among non-Hispanic Black women (6).

During the decade prior to the *Dobbs v. Jackson Women's Health Organization* decision in 2022—a decision that overturned the federal constitutional right to an abortion—states enacted more than 570 abortion restrictions (7). One common category of restrictions, Targeted Regulation of Abortion Providers (TRAP) laws, involved requiring logistically challenging modifications to abortion facilities, such as specific dimensions for room size and corridor width and other modifications not required for or related to patient safety (8). TRAP laws resulted in the closure of almost 100 abortion clinics across the South and Midwest (9). Other restrictions enacted between 2010 and 2020, such as waiting periods, parental notification, and restrictions on insurance coverage, imposed additional burdens and further decreased access to abortion care (10–14). Brown et al. demonstrated that a highly restrictive climate of abortion restrictions, including TRAP laws and other barriers, reduced rates of abortion (15).

Abortion restrictions may have detrimental effects on mental health outcomes among reproductive-aged individuals. The Turnaway Study, a study that assessed the occurrence of mental health diagnoses in women 3 years after they sought an abortion, found that people denied an abortion experienced acute symptoms of anxiety and depression, but after several years these same individuals were no more likely to experience mental health outcomes than their counterparts who received an abortion (16, 17). Zandberg et al. found that the annual rate of suicide among reproductive-aged women was almost six percent higher after enforcement of a state TRAP law, suggesting that state-level abortion restrictions can have population-wide effects on mental health outcomes (18). In a recent large-scale study, Thornburg et al. demonstrated that after *Dobbs*, reproductive-aged women living in states with trigger bans—abortion bans that immediately took effect after *Roe* was overturned by the *Dobbs* decision—experienced greater anxiety and depressive symptoms than those living in states without trigger bans (19).

Given the alarming prevalence of mental health conditions that occur during the perinatal period, affecting one in five birthing people, and the subsequent health consequences such as suicidality and suicide, understanding the impact of abortion restrictions on mental health during pregnancy and postpartum is necessary to support birthing people in times of crisis. Whether abortion restrictions are associated with precursors to suicide during the perinatal period, including mental health crises such as suicidality or self-harm, remains unknown. It is also unknown if trends in suicidality and self-harm differed by state abortion restrictiveness prior to the enactment of post-*Dobbs* abortion bans or near bans. Clarifying the relationship between state abortion restrictiveness and mental health represents an important and timely goal as researchers evaluate the best analytic approach to quantify the impact of abortion bans on health outcomes*.* Furthermore, understanding these relationships is also important information for policymakers and clinicians seeking to mitigate the consequences of policies that restrict access to abortion services.

Accordingly, this study sought to (1) describe trends in suicidality and self-harm by state abortion restrictiveness in the period leading up to *Dobbs* and (2) quantify the association between state abortion restrictiveness and suicidality or self-harm rates in the year before and following delivery. We conducted our analysis in a large population of individuals with employer-sponsored health insurance (ESI) with documented live birth. Understanding the baseline trends and relationships between state-level abortion restrictiveness and mental health outcomes provides a basis for future studies estimating the impact of restrictive reproductive health policies on mental health.

## Materials and methods

### Data source

This analysis is part of the Maternal Behavioral Health Policy Evaluation (MAPLE) study, a retrospective, observational cohort study of the impact of policy changes on mental health during the perinatal period (6). For this paper, we conducted a cross-sectional analysis to quantify the relationship between state-level abortion restrictions and a diagnosis of suicidality or self-harm in the 12 months before or 12 months after delivery among commercially insured in the U.S. between 2010 and 2020 using Optum's de-identified Clinformatics® Data Mart Database (CDM). CDM is derived from a database of de-identified administrative health claims for members of large commercial and Medicare Advantage health plans. These data consist of medical and pharmacy claims including diagnoses and procedures, hospitalizations, outpatient visits, and patient demographic characteristics and do not include medical record information. The University of Michigan Institutional Review Board exempted this study because it analyzes only de-identified data (HUM00240001).

Our analytic sample consisted of individuals aged 15–44 years with a documented live birth between January 1, 2010, and December 31, 2020. We further restricted our sample to those with continuous plan enrollment for at least 12 months before or after delivery. We excluded individuals enrolled in more than one health plan within the two-year observation period. After applying these inclusion and exclusion criteria, the final analytic sample consisted of 610,177 deliveries.

We identified delivery hospitalizations using standardized International Classification of Disease-9th and 10th Revision-Clinical Modification (ICD-9-CM and ICD-10-CM) diagnosis and procedure codes, shown in Supplementary Table S1. We used live births as our unit of analysis; therefore, a person could appear in the dataset more than once during the ten-year observation period. However, we excluded the second birth for any enrollee with more than one birth during a calendar year.

### Outcome variable

Our primary outcome was suicidality or self-harm during the 12 months before or after birth. We identified suicidality using ICD-9-CM or ICD-10-CM diagnostic codes for suicidal ideation or intentional self-harm found in either outpatient or inpatient claims during the perinatal period (12 months before and following a birth). A list of diagnostic codes for suicidality appears in Supplementary Table S2.

### Abortion restrictiveness

Our primary independent variable was state-level abortion restrictiveness. We used a modified version of the Guttmacher Institute's abortion restriction index, which ranges from 1 to 10, and represents a count of the number of abortion restriction categories enacted or in effect in each state by year (see Supplementary Material for a list of categories). We obtained archived yearly data from the Guttmacher Institute in personal communication for 2010–2020. During exploratory work, we examined the relationship between the full index and our outcomes, as well as changes in the index by state and year. We found that within-state abortion restrictions remained relatively stable during our study period.

**Figure 1:**
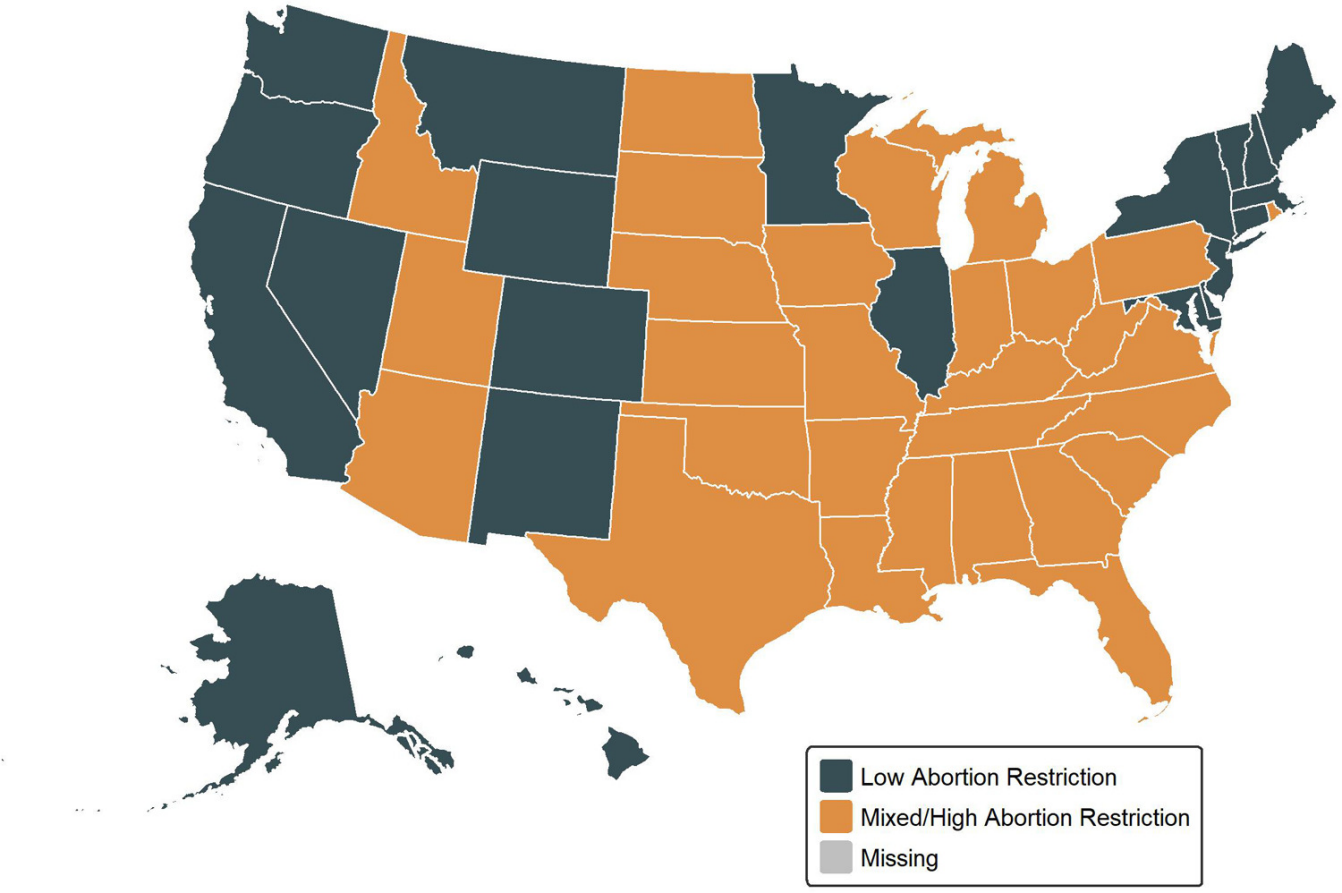
State abortion restrictiveness classification by abortion restrictiveness group (modified guttmacher abortion restrictiveness index).

Based on our exploratory work, we modified the index and grouped states into three categories: (1) low restrictiveness, with 3 or fewer categories of restrictions for at least 8 months of the study period (*n* = 21), (2) high restrictiveness, with 7 or more categories of restrictions for at least 8 months of the study period (*n* = 15), and (3) mixed restrictiveness (all other states, *n* = 14). Initially, we examined trends in the mixed restrictiveness states separately; however, once we observed that outcomes in these states were similar to the outcomes in highly restrictive states, we grouped the mixed states with highly restrictive states. We conducted a sensitivity analysis excluding the 14 mixed restrictiveness states from our analysis, which did not alter our findings (Supplementary Table S5). Our final state categorization appears in Figure 1. A description of our exploratory work and state scores by year appears in detail in Supplementary Table S3.

### Statistical approach

We used summary statistics, such as means, standard deviations, and proportions, to describe our study cohort, overall and by state-level abortion restrictiveness category. We implemented unadjusted and adjusted mixed effects regression models to assess the relationship between state-level abortion restrictiveness and suicidality. We used live births as the unit of analysis and clustered the models by state. All models included year as a covariate to account for the temporal changes, including that suicidality diagnoses have increased over time (6, 20).

For our adjusted models, we assessed individual-level covariates available in our data that were associated with suicidality in prior research: age and race (6). These covariates indicate whether population differences may explain outcomes. We grouped individuals into three age groups, 15–24, 25–34, and 35–44, and used race/ethnicity categories of Asian, Black, Hispanic, White, and unknown (the categories available in CDM's claims). We could not run a model using both age and race as covariates due to small counts of the outcome. We considered an additional model interacting state policy with time to examine the differences in trends in suicidality over time between restrictiveness groups; however, this model did not converge due to a small number of events. For a sensitivity analysis, we examined rates of suicidality by abortion restrictiveness category among birthing people ages 15–24, which was the age group with the largest increase in suicidality over the study period (6).

We conducted analyses using SAS, Version 9.4 (SAS Institute, Cary NC) for data management, summary statistics, and modeling. We used R, Version 4.3.2 to create figures. We used two-sided statistical tests at the 0.05 significance level to assess significance where applicable.

## Results

We identified 610,177 deliveries among enrollees with continuous enrollment 12 months before and after birth who delivered between 2010 and 2020. Birthing people residing in states with high abortion restrictiveness were younger (12.83% of birthing people were ages 15–24 compared to 7.85% in low-restrictiveness states) and more likely to be Black (10.43% compared to 6.03% in low-restrictiveness states). Characteristics of our sample appear in Table 1.

**Table 1:** Characteristics of study sample, overall and by abortion restrictiveness group, 2010–2020.

Total deliveries	Overall (610,177)	Low (216,517)	High (393,660)
Age	N (%)	N (%)	N (%)
15–24	67,501 (11.06%)	16,998 (7.85%)	50,503 (12.83%)
25–34	367,171 (60.17%)	124,847 (57.66%)	242,324 (61.56%)
35–44	175,505 (28.76%)	74,672 (34.49%)	100,833 (25.61%)
Race/ethnicity			
White	398,316 (65.28%)	133,063 (61.46%)	265,253 (67.38%)
Black	54,112 (8.87%)	13,058 (6.03%)	41,054 (10.43%)
Hispanic	80,325 (13.16%)	31,013 (14.32%)	49,312 (12.53%)
Asian	48,585 (7.96%)	27,412 (12.66%)	21,173 (5.38%)
Unknown^a^	28,839 (4.73%)	11,971 (5.53%)	16,868 (4.28%)

^a^ Race/ethnicity was missing in source data.

**Figure 2:**
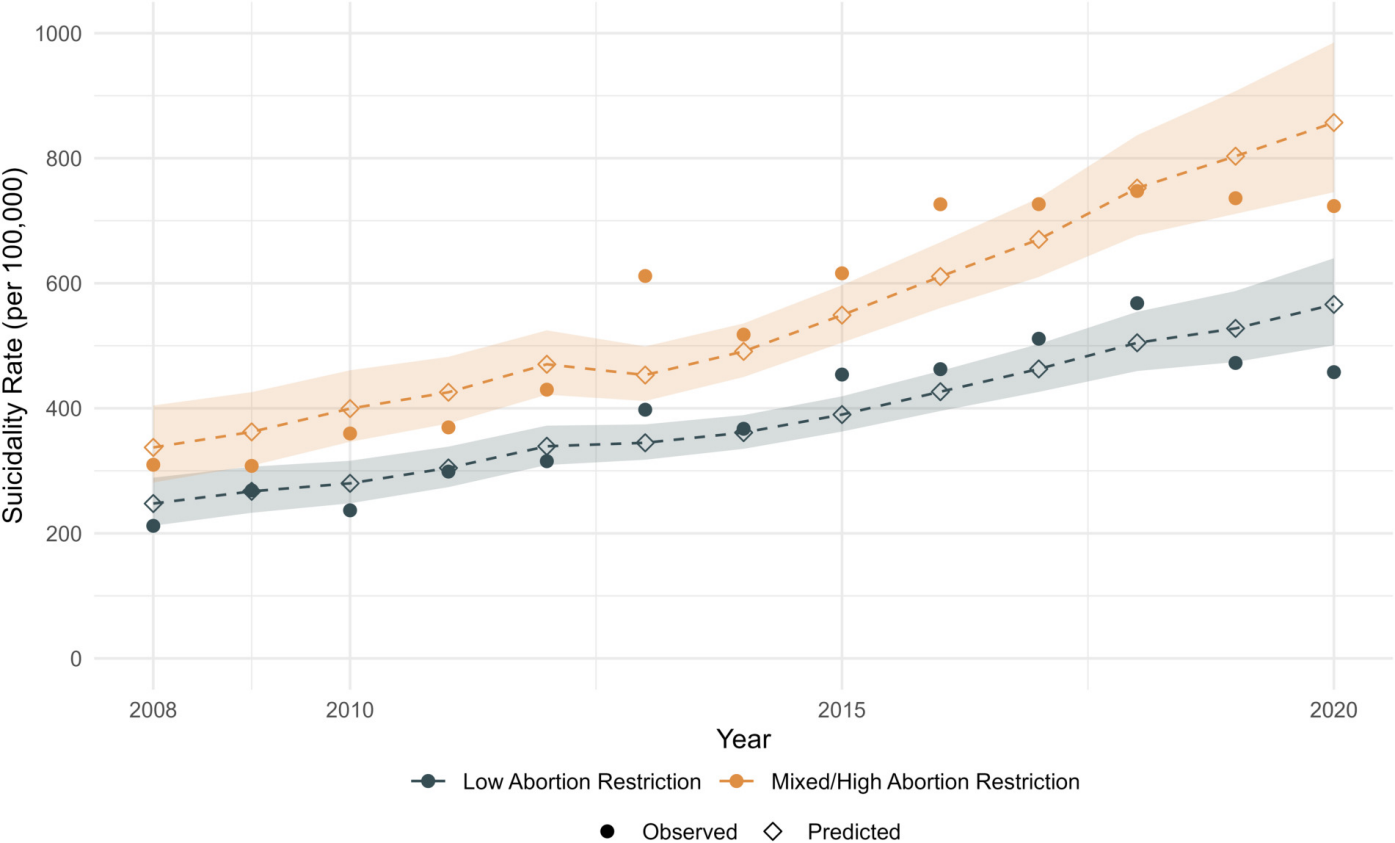
Observed and predicted trends in suicidality in states with low and mixed/high abortion restrictions, 2010-2020. Shaded area represents 95% confidence interval.

Table 2 shows the results from the logistic regression models predicting suicidality. In Model 1, birthing people living in states with high restrictiveness were 1.38 times more likely to experience suicidality than those living in states with low restrictiveness, controlling for temporal trends (95% CI: 1.10–1.73). Suicidality increased in both groups over time, shown in Figure 2.

**Table 2:** Unadjusted and adjusted models of the odds of suicidality by abortion restrictiveness group, 2010–2020.

Covariate	Odds (95% CI)		
	Model 1	Model 2	Model 3
Residence in state with high level of abortion restrictions	1.38 (1.10, 1.73)*	1.17 (0.99, 1.37)	1.31 (1.05, 1.64)*
Year	1.07 (1.06, 1.09)*	1.08 (1.07, 1.09)*	1.07 (1.06, 1.09)*
Age			
35–44	–	Reference	–
25–34	–	7.78 (7.01, 8.62)*	–
15–24	–	1.02 (0.91, 1.13)	–
Race			
White	–	–	Reference
Black	–	–	1.64 (1.47, 1.82)*
Asian	–	–	0.56 (0.47, 0.68)*
Hispanic	–	–	0.98 (0.88, 1.11)
Unknown	–	–	0.96 (0.81, 1.15)

* *p* < 0.05.

We then added age group and race as covariates into two additional models. After adjusting for age, those living in highly restrictive states were no longer significantly more likely to have a suicidality or self-harm diagnosis (adjOR = 1.17, 95% CI: 0.99–1.37). In this model, younger birthing people (ages 15–24) were 7.78 times more likely to experience suicidality or self-harm compared to birthing people ages 35–44 (95% CI: 7.01–8.62, Model 2). In the separate model adjusting for race, we found that birthing people living in highly restrictive states were 1.31 times more likely to experience suicidality (95% CI: 1.05–1.64). Odds of suicidality also varied by race; Black birthing people were 1.64 times more likely to experience suicidality than White birthing people (95% CI: 1.47–1.82), and Asian birthing people were 0.56 times less likely to experience suicidality than White birthing people (95% CI: 0.47–0.68, Model 3).

Since suicidality rates were highest and increased the greatest among the youngest age group, we ran a model restricted to ages 15–24. Among this age group, rates of suicidality increased by about 10% each year, with no significant difference between those living in states with high restrictiveness and those living in states with low restrictiveness (see Supplementary Table S4 and Supplementary Figure S2).

To test the sensitivity of our state categorization, we ran a model excluding the 12 states that had variations in abortion restrictiveness between 2010 and 2020. We found similar results to our main analysis: residing in a state with more abortion restrictions remained not significantly associated with a higher likelihood of suicidality, and birthing people ages 15–24 were substantially more likely to experience suicidality compared to those ages 35–44 (adjOR 7.96, 95% CI: 7.04–9.00) (see Supplementary Table S5).

## Discussion

We found that in the years prior to the *Dobbs* decision of 2022, birthing people living in states with high abortion restrictiveness were more likely to experience suicidality or self-harm than those living in states with low or mixed abortion restrictiveness. However, this finding appears to be driven by age differences rather than by abortion restrictiveness.

In our analysis of birthing people with ESI, age and racial differences largely explained state differences in suicidality rates during the perinatal period, with age appearing to explain most of the differences. This observation aligns with prior research, including an analysis conducted in a similar population, demonstrating that younger and Black birthing people experience higher rates of suicidality compared to older individuals or those of other racial backgrounds (6). Another analysis consistent with this observation found that suicidal ideation, suicide attempts, and non-suicidal self-harm were greatest among hospitalized Non-Hispanic Black pregnant women (21). These findings represent crucial information for future studies isolating the impact of abortion bans on mental health.

Our finding that birthing people in the youngest age group (15–24 years old) were more than 7 times more likely to experience suicidality compared to older birthing people adds to the evidence of a growing youth mental health crisis (20, 22). In 2020, the American Academy of Pediatrics, American Academy of Child and Adolescent Psychiatry, and Children's Hospital Association declared an emergency in child and adolescent mental health as rates of childhood mental health concerns and suicide increased steadily between 2010 and 2020, with suicide the second leading cause of death for youth ages 10-24 in 2018 (23).

Previous research suggests that adolescents remain especially vulnerable to barriers to abortion access such as travel and financial challenges and parental notification and consent requirements that also limit access to medication abortion (24). Taken together, our finding suggests that future researchers should take care to understand the special considerations of this population with respect to mental health and abortion restrictions. Future work should examine the impact of abortion restrictions on adolescent mental health specifically, including parental consent laws. Other marginalized groups may also require special consideration, as these findings also align with previous research showing increases in suicidality among all birthing people, younger people, and Black individuals (6, 20, 22, 25).

We found higher suicidality among Black birthing individuals when compared to White individuals, which may be explained in part by systemic racism. For instance, prior literature suggests that symptomatic Black individuals are less likely to receive perinatal mood and anxiety disorder diagnoses and treatment compared to White individuals (26). Further, healthcare practitioners may not identify as many Black individuals in mental health crisis, and Black individuals may not receive as much treatment, prior to an acute crisis (27), when compared to White individuals.

Our findings also contrast with a recent study demonstrating the association between enactment of abortion restrictions and increases in completed suicide among reproductive age individuals (18). Differences in study design and outcomes likely explain these divergent findings.

Our study sample included a smaller and relatively higher-income population of individuals with ESI. This population may have more access to resources to overcome barriers to services. Future studies should examine the experiences of lower-income individuals, including those with Medicaid, because these individuals may face more barriers. Our study also examined a shorter and different timespan (2010–2020 in the present study compared with 1974–2016 in the Zandberg study). Individuals within more restrictive states may have different options to seek abortion care within the different timespans of these studies, which may differentially impact mental health.

Increased enactment of abortion restrictions occurred during the study period of 2010–2020 compared to prior years, though more substantial restrictions followed the *Dobbs* decision of 2022. For example, data suggest that self-managed abortions, out-of-state travel for abortions, and medication abortion have all increased since the *Dobbs* decision (28). As of November 2024, 13 states have active total abortion bans, and 6 additional states set gestational limits for abortion at 12 weeks or less (29). In 2023, nearly 25 million American women of childbearing age lived in a state with abortion access curtailed after the *Dobbs* ruling (30). We expect that post-*Dobbs* abortion restrictions will have a much greater impact on abortion access, and future research should include examinations of mental health effects of these restrictions.

### Strengths and limitations

The strengths of this study include our large, national sample of diverse individuals which allows us to examine a relatively rare outcome. Our analysis also had several notable limitations. First, we could not use a quasi-experimental method, such as difference-in-difference analysis, due to the infrequency of our outcome and the within-state stability of our exposure (abortion restrictiveness) in our observation period. These findings cannot establish causality. Additional limitations inherent to insurance claims data mean that we are only able to observe covered services; therefore some individuals in mental health crisis may not be identified. These data also do not contain individual factors that may be important risk factors, including marital status or pregnancy wantedness. We also acknowledge that this study was conducted among individuals covered by ESI (employees and their dependents), a more advantaged population that may experience less impact of abortion restrictions than other populations (e.g., Medicaid). Finally, this study was conducted prior to the widespread enactment of abortion bans or near bans. Therefore, it does not include the most restrictive abortion policies.

Since the outcome of interest, suicidality, remains relatively uncommon (ranging from approximately 300 per 100,000 births in 2010–600 per 100,000 births in 2020, as shown in Figure 2), we could not conduct models that included both race and age. We also could not examine other sociodemographic factors, such as marital status or income, which may also contribute to risk. Future research should also examine differences within at-risk groups to determine if restricting access to abortion impacts some groups of birthing people differently from others.

## Conclusion

In the years prior to the *Dobbs* decision of 2022, which ushered in total abortion bans in 13 states and gestational limits for abortion of 12 weeks or less in 6 states, commercially insured birthing people in highly restrictive states experienced increased suicidality and self-harm, when compared to those in less restrictive states. However, these differences appeared to occur largely in relation to changing demographic characteristics, such as age and race. As we continue to monitor health outcomes related to the recent enactment of the most severe category of restriction (e.g., bans), these findings remain crucial to recognize the impact of barriers to abortion care on mental health. Future research should also examine mental health outcomes among other, and perhaps more vulnerable, populations.

## Data Availability

The data analyzed in this study is subject to the following licenses/restrictions: the data can be purchased from Optum and obtained with a data use agreement. We have included coding used in our Supplementary Materials. Requests to access these datasets should be directed to https://business.optum.com/en/data-analytics/life-sciences/real-world-data.html.
