# Supplementary material for: Association between abortion restrictiveness and suicidality among birthing people in the United States 2010 to 2020
Source: Front Reprod Health. 2025 Aug 29;7:1553493. doi: 10.3389/frph.2025.1553493 (PMC12426194; doi:10.3389/frph.2025.1553493)
Supplement: Supplementary file 1 [file Table1.docx]

**Supplementary Material**

**Supplementary Table 1. Diagnosis and procedure codes identifying live births**

| Delivery | Code type | Code List |
| --- | --- | --- |
| Cesarean delivery | ICD-9-CM | 740, 741, 742, 744, 7499 |
| Cesarean delivery | ICD-10-CM | 10D00Z0, 10D00Z1, 10D00Z2 |
| Cesarean delivery | DRG | 370, 371, 765, 766, 540, 5401, 5402, 5403, 5404 |
| Cesarean delivery | CPT | 59510, 59514, 59515, 59618, 59620, 59622 |
| Vaginal delivery | ICD-10-CM | 10D07Z3, 10D07Z4, 10D07Z5, 10D07Z6, 10D07Z7, 10D07Z8, 10E0XZZ |
| Vaginal delivery | DRG | 372, 373, 374, 375, 767 768, 774, 775, 541, 542, 560, 5411, 5412, 5413, 5414, 5421, 5422, 5423, 5424, 5601, 5602, 5603 |
| Vaginal delivery | CPT | 59400, 59409, 59410, 59610, 59612, 59614 |
| Preterm birth | ICD-9 | O601, O6010, O6010X0, O6010X1, O6010X2, O6010X3, O6010X4, O6010X5, O6010X9, O6012, O6012X0, O6012X1, O6012X2, O6012X3, O6012X4, O6012X5, O6012X9, O6013, O6013X0, O6013X1, O6013X2, O6013X3, O6013X4, O6013X5, O6013X9, O6014, O6014X0, O6014X1, O6014X2, O6014X3, O6014X4, O6014X5, O6014X9 |
| Preterm birth | ICD-10 | 64420, 64421 |

**Supplementary Table 2. Diagnosis codes identifying suicidality**

| Code type | Code List |
| --- | --- |
| ICD-9 | E9500, E9501, E9502, E9503, E9504, E9505, E9506, E9507, E9508, E9509, E9510, E9511, E9518, E9520, E9521, E9528, E9529, E9530, E9531, E9538, E9539, E954, E9550, E9551, E9552, E9553, E9554, E9555, E9556, E9557, E9559, E956, E9570, E9571, E9572, E9579, E9580, E9581, E9582, E9583, E9584, E9585, E9586, E9587, E9588, E9589, E959 |
| ICD-10 | R45851, R4586, T1491, T1491XA, T1491XD, T1491XS, T40995A, T40995D, T40995S, T40996A, T40996D, T40996S, X710XXA, X710XXD, X710XXS, X711XXA, X711XXD, X711XXS, X712XXA, X712XXD, X712XXS, X713XXA, X713XXD, X713XXS, X718XXA, X718XXD, X718XXS, X719XXA, X719XXD, X719XXS, X72XXXA, X72XXXD, X72XXXS, X730XXA, X730XXD, X730XXS, X731XXA, X731XXD, X731XXS, X732XXA, X732XXD, X732XXS, X738XXA, X738XXD, X738XXS, X739XXA, X739XXD, X739XXS, X7401XA, X7401XD, X7401XS, X7402XA, X7402XD, X7402XS, X7409XA, X7409XD, X7409XS, X748XXA, X748XXD, X748XXS, X749XXA, X749XXD, X749XXS, X75XXXA, X75XXXD, X75XXXS, X76XXXA, X76XXXD, X76XXXS, X770XXA, X770XXD, X770XXS, X771XXA, X771XXD, X771XXS, X772XXA, X772XXD, X772XXS, X773XXA, X773XXD, X773XXS, X778XXA, X778XXD, X778XXS, X779XXA, X779XXD, X779XXS, X780XXA, X780XXD, X780XXS, X781XXA, X781XXD, X781XXS, X782XXA, X782XXD, X782XXS, X788XXA, X788XXD, X788XXS, X789XXA, X789XXD, X789XXS, X79XXXA, X79XXXD, X79XXXS, X80XXXA, X80XXXD, X80XXXS, X810XXA, X810XXD, X810XXS, X811XXA, X811XXD, X811XXS, X818XXA, X818XXD, X818XXS, X820XXA, X820XXD, X820XXS, X821XXA, X821XXD, X821XXS, X822XXA, X822XXD, X822XXS, X828XXA, X828XXD, X828XXS, X830XXA, X830XXD, X830XXS, X831XXA, X831XXD, X831XXS, X832XXA, X832XXD, X832XXS, X838XXA, X838XXD, X838XXS, Z915 |

**Supplementary Figure 1. Suicidality rate by Guttmacher Institute’s abortion restrictiveness index.**


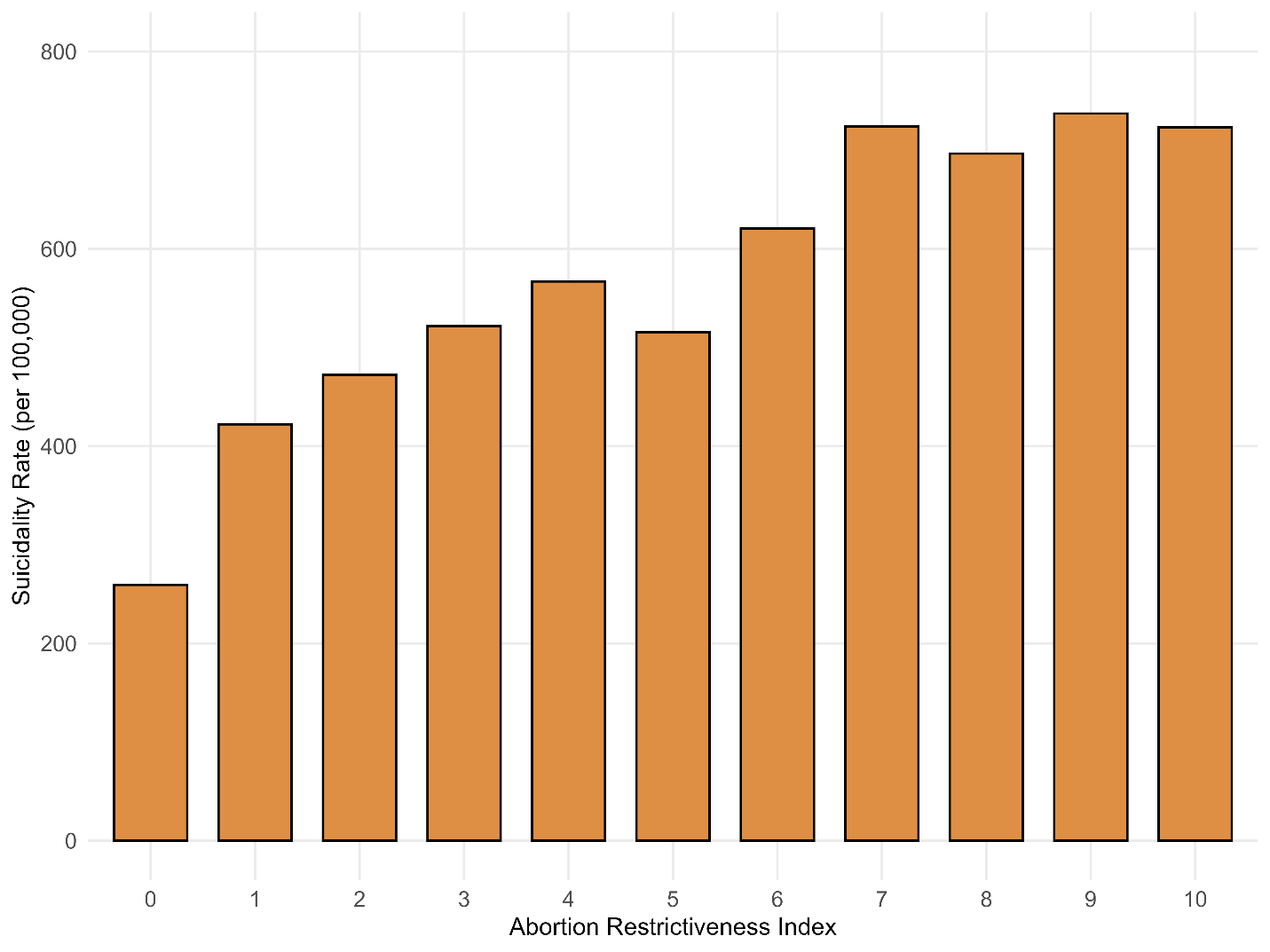


We used 2010-2020 data from the Guttmacher Institute’s abortion restriction index to create our categories of abortion restrictiveness used in our analyses (obtained from personal communication). We included the following ten categories of restrictions:

1. Trigger laws
2. 20 week ban or unconstitutional post-viability abortion restriction
3. Inaccurate or misleading counseling
4. In-person counseling (two trips)
5. Ultrasound mandated
6. Abortion coverage restricted (all, exchange only, state employee)
7. Abortion coverage restricted in Medicaid
8. Medication abortion restriction (telemedicine, FDA protocol)
9. Parental involvement (notice, consent or both)
10. TRAP (clinic regulations and/or admitting privileges)

First, to identify appropriate boundaries for our restrictiveness groups, we examined the relationship between the AGI index and rates of suicidality. (Shown in Supplementary Figure 1). We limited to state-year combinations with at least one suicidality or self-harm event, given low frequency of the outcome measure, and included weights accounting for differences in state-level penetration of CDM in the delivery population. Based on this exploratory analysis, we defined low restrictiveness as 0-3 categories of restrictions for 8 or more years of the study period; high restrictiveness as 7-10 categories of restrictions for 8 or more years of the study period; and mixed restrictiveness as states that do not meet either category (i.e., have a moderate level of restrictions or move from low to high restrictiveness during the study period). This decision was based on the observed relationship between the index and our outcomes, the distribution of states in these categories, and the within state variation during our observation period, which is described below.

Supplementary Table 3 demonstrates each state’s score by year. After examining within state variation in scores during the observation period, we found that most states were fairly stable in their restrictiveness scores – either on the low or high ends of the index. For instance, between 2012 and 2020 only 14 states had a 3 or more point change in their index.

**Supplementary Table 3. Index of abortion restrictiveness laws and categorization**

| **States** | **2010** | **2011** | **2012** | **2013** | **2014** | **2015** | **2016** | **2017** | **2018** | **2019** | **2020** |
| --- | --- | --- | --- | --- | --- | --- | --- | --- | --- | --- | --- |
| **Low restrictiveness** | | | | | | | | | | | |
| Connecticut | 0 | 0 | 0 | 0 | 0 | 0 | 0 | 0 | 0 | 0 | 0 |
| Hawaii | 0 | 0 | 0 | 0 | 0 | 0 | 0 | 0 | 0 | 0 | 0 |
| New Jersey | 0 | 0 | 0 | 0 | 0 | 0 | 0 | 0 | 0 | 0 | 0 |
| New Mexico | 0 | 0 | 0 | 0 | 0 | 0 | 0 | 0 | 0 | 0 | 0 |
| Oregon | 0 | 0 | 0 | 0 | 0 | 0 | 0 | 0 | 0 | 0 | 0 |
| Vermont | 0 | 0 | 0 | 0 | 0 | 0 | 0 | 0 | 0 | 0 | 0 |
| Washington | 0 | 0 | 0 | 0 | 0 | 0 | 0 | 0 | 0 | 0 | 0 |
| California | 1 | 1 | 1 | 0 | 0 | 0 | 0 | 0 | 0 | 0 | 0 |
| Maine | 1 | 1 | 1 | 1 | 1 | 1 | 1 | 1 | 1 | 0 | 0 |
| New York | 1 | 1 | 1 | 1 | 1 | 1 | 1 | 1 | 1 | 0 | 0 |
| Maryland | 1 | 1 | 1 | 1 | 1 | 1 | 1 | 1 | 1 | 1 | 1 |
| Alaska | 1 | 2 | 2 | 2 | 2 | 2 | 2 | 2 | 1 | 1 | 1 |
| Montana | 1 | 1 | 2 | 2 | 2 | 2 | 2 | 2 | 2 | 2 | 2 |
| New Hampshire | 1 | 1 | 2 | 2 | 2 | 2 | 2 | 2 | 2 | 2 | 2 |
| Delaware | 2 | 2 | 2 | 2 | 2 | 2 | 2 | 2 | 2 | 2 | 2 |
| Massachusetts | 2 | 2 | 2 | 2 | 2 | 2 | 2 | 2 | 2 | 2 | 2 |
| Minnesota | 2 | 2 | 2 | 2 | 2 | 2 | 2 | 2 | 2 | 2 | 2 |
| Nevada | 2 | 2 | 2 | 2 | 2 | 2 | 2 | 2 | 2 | 2 | 2 |
| Illinois | 2 | 2 | 2 | 3 | 3 | 3 | 3 | 3 | 1 | 1 | 1 |
| Colorado | 3 | 3 | 3 | 3 | 3 | 3 | 3 | 3 | 3 | 3 | 3 |
| Wyoming | 3 | 3 | 3 | 3 | 3 | 3 | 3 | 3 | 3 | 3 | 3 |
| **Mixed restrictiveness** | | | | | | | | | | | |
| West Virginia | 2 | 2 | 2 | 2 | 2 | 3 | 3 | 4 | 5 | 5 | 5 |
| Iowa | 3 | 3 | 3 | 3 | 3 | 3 | 3 | 4 | 4 | 4 | 4 |
| Rhode Island | 4 | 4 | 4 | 4 | 4 | 4 | 4 | 4 | 4 | 3 | 3 |
| Idaho | 4 | 4 | 4 | 4 | 4 | 5 | 5 | 4 | 4 | 4 | 5 |
| Georgia | 4 | 4 | 4 | 4 | 5 | 5 | 5 | 5 | 5 | 5 | 5 |
| Pennsylvania | 5 | 5 | 5 | 5 | 5 | 5 | 5 | 5 | 5 | 5 | 5 |
| Kentucky | 4 | 4 | 4 | 4 | 4 | 4 | 5 | 6 | 6 | 8 | 10 |
| South Carolina | 5 | 5 | 5 | 5 | 5 | 5 | 6 | 6 | 6 | 6 | 6 |
| North Carolina | 4 | 5 | 5 | 6 | 6 | 6 | 6 | 6 | 6 | 6 | 6 |
| Tennessee | 3 | 5 | 5 | 5 | 5 | 6 | 6 | 6 | 6 | 7 | 8 |
| Florida | 4 | 6 | 6 | 6 | 6 | 6 | 6 | 6 | 6 | 6 | 6 |
| Wisconsin | 3 | 3 | 5 | 6 | 6 | 6 | 7 | 7 | 7 | 7 | 7 |
| Michigan | 5 | 5 | 5 | 6 | 7 | 7 | 7 | 7 | 7 | 6 | 6 |
| Arkansas | 4 | 4 | 4 | 6 | 6 | 8 | 8 | 8 | 8 | 9 | 10 |
| **High restrictiveness** | | | | | | | | | | |  |
| Virginia | 4 | 5 | 7 | 7 | 7 | 7 | 7 | 7 | 7 | 7 | 6 |
| Alabama | 5 | 5 | 6 | 7 | 7 | 7 | 7 | 7 | 7 | 7 | 7 |
| Ohio | 6 | 6 | 6 | 7 | 7 | 7 | 7 | 7 | 7 | 7 | 7 |
| Nebraska | 5 | 7 | 7 | 7 | 7 | 7 | 7 | 7 | 7 | 7 | 7 |
| Utah | 6 | 7 | 7 | 7 | 7 | 7 | 7 | 7 | 7 | 7 | 7 |
| Kansas | 2 | 7 | 7 | 8 | 8 | 8 | 8 | 8 | 8 | 8 | 8 |
| Oklahoma | 5 | 7 | 7 | 7 | 8 | 8 | 8 | 8 | 8 | 8 | 8 |
| North Dakota | 6 | 7 | 7 | 7 | 8 | 8 | 8 | 8 | 8 | 8 | 8 |
| Missouri | 5 | 7 | 7 | 8 | 8 | 8 | 8 | 7 | 8 | 9 | 9 |
| Texas | 5 | 5 | 7 | 8 | 8 | 8 | 8 | 9 | 9 | 9 | 9 |
| Arizona | 2 | 5 | 8 | 9 | 9 | 9 | 9 | 9 | 9 | 9 | 9 |
| Indiana | 5 | 7 | 7 | 9 | 9 | 9 | 9 | 9 | 9 | 9 | 9 |
| South Dakota | 5 | 7 | 8 | 8 | 9 | 9 | 9 | 9 | 9 | 9 | 9 |
| Mississippi | 8 | 8 | 8 | 9 | 10 | 10 | 10 | 10 | 10 | 10 | 10 |
| Louisiana | 7 | 8 | 9 | 10 | 10 | 10 | 10 | 10 | 10 | 10 | 10 |

Notes: data compiled by the Guttmacher Institute and categorized by the authors. The index indicates the number of categories of laws restricting abortion within a state.

**Supplementary Table 4. Sensitivity analysis of the unadjusted model among birthing people aged 15-24**

|  | Odds (95% CI) |
| --- | --- |
| Residence in state with high level of abortion restrictions | 1.03 (0.83, 1.27) |
| Year | 1.10 (1.08, 1.12)* |

* p < 0.05

**Supplementary Figure 2. Suicidality rate by abortion restrictiveness group over time age 15-24, 2010-2020**


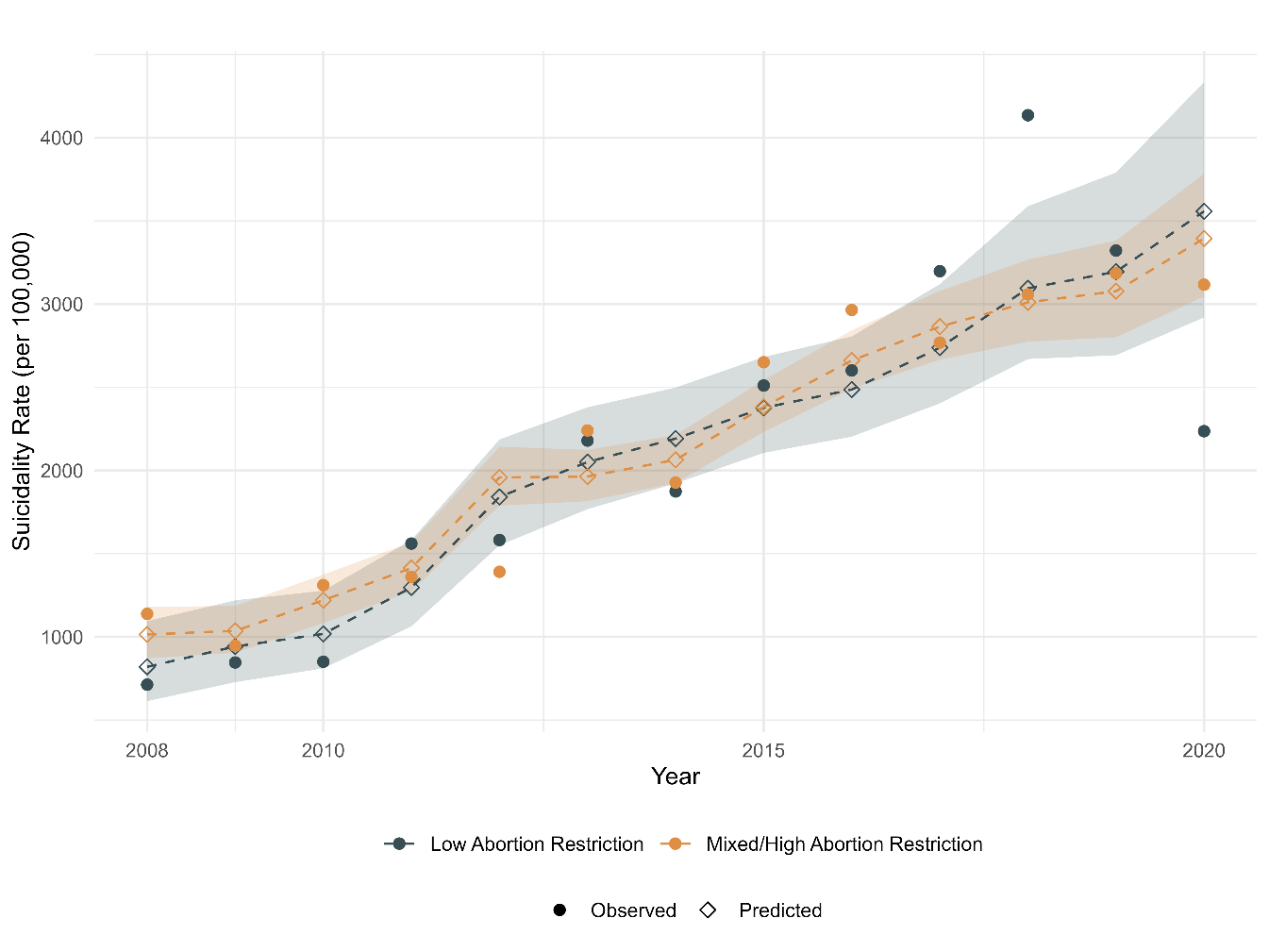


**Supplementary Table 5. Sensitivity analysis of the age-adjusted model excluding states classified as “mixed”**

|  | Odds (95% CI) |
| --- | --- |
| Residence in state with high level of abortion restrictions | 0.83 (0.67, 1.02) |
| Year | 1.080 (1.066, 1.09)* |
| Age |  |
| 35-44 | Reference |
| 25-34 | 1.06 (0.94, 1.21) |
| 15-24 | 7.96 (7.04, 9.00)* |

* p < 0.05

Excluded states: GA, IA, ID, MI, NC, NH, PA, RI, SC, VA, WV, WY
